# Trends in survival after a diagnosis of heart failure in the United Kingdom 2000-2017: population based cohort study

**DOI:** 10.1136/bmj.l223

**Published:** 2019-02-13

**Authors:** Clare J Taylor, José M Ordóñez-Mena, Andrea K Roalfe, Sarah Lay-Flurrie, Nicholas R Jones, Tom Marshall, F D Richard Hobbs

**Affiliations:** 1 Nuffield Department of Primary Care Health Sciences, University of Oxford, Oxford OX2 6GG, UK; 2 Institute of Applied Health Research, University of Birmingham, Birmingham, UK

## Abstract

**Objectives:**

To report reliable estimates of short term and long term survival rates for people with a diagnosis of heart failure and to assess trends over time by year of diagnosis, hospital admission, and socioeconomic group.

**Design:**

Population based cohort study.

**Setting:**

Primary care, United Kingdom.

**Participants:**

Primary care data for 55 959 patients aged 45 and over****with a new diagnosis of heart failure and 278 679 age and sex matched controls in the Clinical Practice Research Datalink from 1 January 2000 to 31 December 2017 and linked to inpatient Hospital Episode Statistics and Office for National Statistics mortality data.

**Main outcome measures:**

Survival rates at one, five, and 10 years and cause of death for people with and without heart failure; and temporal trends in survival by year of diagnosis, hospital admission, and socioeconomic group.

**Results:**

Overall, one, five, and 10 year survival rates increased by 6.6% (from 74.2% in 2000 to 80.8% in 2016), 7.2% (from 41.0% in 2000 to 48.2% in 2012), and 6.4% (from 19.8% in 2000 to 26.2% in 2007), respectively. There were 30 906 deaths in the heart failure group over the study period. Heart failure was listed on the death certificate in 13 093 (42.4%) of these patients, and in 2237 (7.2%) it was the primary cause of death. Improvement in survival was greater for patients not requiring admission to hospital around the time of diagnosis (median difference 2.4 years; 5.3 *v* 2.9 years, P<0.001). There was a deprivation gap in median survival of 0.5 years between people who were least deprived and those who were most deprived (4.6 *v* 4.1 years, P<0.001).

**Conclusions:**

Survival after a diagnosis of heart failure has shown only modest improvement in the 21st century and lags behind other serious conditions, such as cancer. New strategies to achieve timely diagnosis and treatment initiation in primary care for all socioeconomic groups should be a priority for future research and policy.

## Introduction

Heart failure is a common and costly clinical syndrome, but it can be treated effectively.^1^ ^2^ A rise in cardiovascular risk factors, improved survival from ischaemic heart disease, and population ageing have contributed to a sustained increase in prevalence.^3^ Recent analysis of primary care data in the United Kingdom found the absolute number of people living with heart failure increased by 23% between 2002 and 2014 from 750 125 to 920 616 (1.4% of the population).^4^ The global economic cost of heart failure is estimated at US$108bn (£82.4bn; €94.5bn) per year, comprising direct costs to healthcare systems and indirect costs to society through loss of productivity. The greatest expenditure is in the last three months of life.^5^ ^6^ Pharmacological treatments, devices, and exercise based rehabilitation can improve outcomes for patients, particularly those with heart failure with reduced ejection fraction, but diagnosis is crucial to allow timely initiation of evidence based treatments.

Reliable and contemporary survival estimates are important for any long term condition at the population level to monitor trends in prognosis and to commission appropriate services. At a patient level, these estimates allow informed discussions and shared decision making about treatment options and advanced care planning.^7^ Previous prognostic studies in heart failure have used data from hospital inpatients or screening studies to report survival. People with established heart failure who need to be admitted to hospital for acute decompensation have high mortality rates; up to one in six patients die during admission or within 30 days after discharge.^8^ ^9^ ^10^ Survival for screen detected heart failure is more favourable, with around half of all study participants alive at five years.^11^ ^12^ Studies of people diagnosed as having heart failure in a routine community setting are limited and inconsistent, and trends in survival over time are conflicting.^13^ ^14^ ^15^ Analysis of primary care records in The Health Improvement Network showed no change in survival after a diagnosis of heart failure between 1998 and 2012; this finding was received with some concern by the heart failure community and the wider public.^13^ Contemporary data are needed to establish if outlook has improved in recent years and to explore factors associated with worse outcome.

Charities, cardiology organisations, and government bodies have produced regional and country specific guidelines to inform evidence based practice with the aim of improving patient outcomes, but uptake is not universal.^16^ ^17^ The National Heart Foundation of Australia and the National Institute for Health and Care Excellence in England have recently updated their heart failure guidelines with new recommendations. These guidelines include a focus on collaboration between primary and secondary care.^18^ ^19^ However, within healthcare systems, heart failure has not received the same strategic focus and funding resources as other long term conditions.

In this study we aimed to use primary care records, linked to inpatient and mortality data, to report the short term, mid-term, and long term survival rates of people with heart failure in the community; and to examine trends over time by year of diagnosis, hospital admission around the time of diagnosis, and socioeconomic group.

## Methods

### Design and setting

We performed an open matched, retrospective population based cohort study using data from the Clinical Practice Research Datalink (CPRD) for the period from 1 January 2000 to 31 December 2017. CPRD is a primary care database containing electronic patient records from over 700 general practices and is representative of the UK population.^20^ At each consultation, symptoms or diagnoses are entered using a clinical coding system. Clinical observations, laboratory tests, prescriptions, and demographic details also form part of the electronic record.

We included practices in the study that had contributed at least one year of clinical data. Data quality measures included the up-to-standard date (which incorporates death reporting) and the patient acceptable flag. CPRD classifies patient records as “acceptable” for research purposes after a simple quality check to ensure that the records are as accurate and reliable as possible. CPRD data were linked to the Office for National Statistics (ONS) mortality data to provide the date and cause of death. The data were also linked to inpatient Hospital Episode Statistics (HES) to determine hospital admission around the time of diagnosis and the index of multiple deprivation to determine socioeconomic status. The index of multiple deprivation is calculated for the postal district of the individual’s place of residence, and combines information from seven domains (income, employment, education and training, health and disability, crime, barriers to housing and services, and living environment).^21^ The validity and reliability of the index as a measure of socioeconomic status has previously been reported.^22^

### Study population

We used CPRD to extract acceptable patient records of people aged 45 and over, registered at an up-to-standard practice for at least a year between 1 January 2000 and 31 December 2017. Entry criteria to the cohort were a diagnostic code of heart failure in the primary care record and eligibility for HES and ONS linkage.

Patients entered the cohort on the latest of the following dates: 1 January 2000, date of 45th birthday, patient registration date plus one year, practice up-to-standard date plus one year. We excluded patients with a diagnosis of heart failure that occurred before this date. Patients exited the cohort on the earliest of the following dates: 31 December 2017, patient transferred out date, date of death, last date of practice data collection, last date of available linked data.

We extracted demographic variables including age, sex, ethnicity, patient level deprivation (index of multiple deprivation), cardiovascular risk factors, and comorbidities for each participant. Information on cardiovascular risk factors (smoking, blood pressure, cholesterol, and body mass index) was the most recently recorded before the index date (the first date of a recorded heart failure code in the primary care record within the study period). Cardiovascular comorbidities (angina, myocardial infarction, ischaemic heart disease, diabetes, hypertension, stroke, atrial fibrillation, and valve disease) were defined by the presence of a clinical code at any time before the index date.

### Case definition

We defined heart failure cases as people with a new diagnosis of heart failure in their primary care record. The NHS terminology and classifications browser, the Quality and Outcomes Framework guidance, and ICD-10 (international classification of diseases, 10th revision) were used to generate a comprehensive list of terms to code a diagnosis of heart failure (see appendix 1).

We identified the first diagnosis of heart failure in CPRD as the earliest recorded diagnostic code in the general practitioner’s record within the study period (the index date). The time to death was measured from the index date. People with heart failure were matched by age (±5 years) and sex with up to five comparators registered in the same practice on the index date without a diagnosis of heart failure on that date (but they could develop the condition later).

### Database linkage

Linked data were supplied directly by CPRD. The process used was a deterministic matching algorithm, which matched exactly on NHS number and on at least one other identifier (date of birth, sex, and postcode). More than 97% of HES records and 98% of ONS mortality data are successfully matched in this way. 

We used linked HES data to identify people admitted to hospital within three months of diagnosis (inpatient clinical code of heart failure or inpatient echocardiography report). This three month period before or after the heart failure diagnosis was entered in the primary care record was chosen to allow time for flow of information between hospitals and practices.

Cause of death is determined by ONS according to the information provided on the death certificate by the examining doctor and is used in national statistics in the UK. This information is probably the most accurate available.^23^

### Outcomes

The primary outcome measure was death (all cause mortality). We obtained the date and cause of death from ONS mortality data. Secondary outcomes, reported descriptively, included primary cause of death, death due to heart failure (at any position in the cause of death hierarchy), and death due to arrhythmias.

### Statistical analysis

We identified the number of patients with heart failure and presented baseline socioeconomic demographics for patients and matched comparators. In each group, we determined survival rates at one, five, 10, and 15 years overall, for men and women, and for each 10 year age group from age 45 and over. To investigate trends in mortality over time, survival at one, five, and 10 years was determined by year of diagnosis and by hospital admission around the time of diagnosis. We investigated linear trends in survival over time by fitting weighted linear regression of the survival rate to the year of diagnosis in which the weights were inversely proportional to the variance of the survival rate. We examined socioeconomic inequalities by comparing median survival for people who were least deprived and most deprived according to the index of multiple deprivation. We calculated the difference in survival rates between the earliest and most recent years of diagnosis, and determined 95% confidence intervals using the normal distribution. To adjust for any changes in the age or sex structure of the heart failure population over time, we performed an additional sensitivity analysis in which survival rates were directly standardised by age and sex to the 2000 population. Mixed modelling, adjusting for age, sex, and clustering of patients within practices, was used to confirm any observed association between deprivation and survival over time.

We used Kaplan-Meier curves and log rank tests to compare survival in people with and without heart failure, and by sex, age, and index of multiple deprivation. Cox proportional hazards regression analyses assessed the overall effect of heart failure on survival by adjusting for potential confounders. To preserve the matched study design, the initial adjustment for age, sex, practice, and time of diagnosis was performed by stratifying on matched set. Further adjustment allowed for index of multiple deprivation, ethnicity, and cardiovascular risk factors; these risk factors include lifestyle modifiable risk factors (body mass index, smoking status, systolic and diastolic blood pressure, total cholesterol) and medical history (angina, myocardial infarction, ischaemic heart disease, diabetes, hypertension, stroke, atrial fibrillation, valve disease). We tested the proportional hazards assumption by plotting Schoenfeld residuals over time. No clear trends over time were evident for any of the covariates in the model.

There were substantial missing data for cholesterol and body mass index. A comparison of the characteristics of people with and without missing data suggested the data were not missing at random. Therefore, we considered multiple imputation inappropriate and we undertook an alternative approach in which continuous variables were categorised and unrecorded data represented by an additional missing category. We performed a complete case analysis as a sensitivity analysis, with and without cholesterol and body mass index as covariates. Statistical analysis was carried out using R (version 3.5.0), with “survival” and “survminer” packages.^24^ ^25^ ^26^

### Patient and public involvement

We are grateful to our two patient representatives who have heart failure and informed the research question and design for this study. They initially found “heart failure” a frightening term, which suggested an imminent demise, and were surprised to find it was a long term condition which they would learn to live with. They thought that clinical staff inadequately covered the issue of survival following a diagnosis, and they would “like doctors to have the facts” because the prognostic information given to them had been very limited. We plan to disseminate the findings of this research to patients, carers, heart failure charities, research funders, and policy makers using our social media platforms. We will use the hashtag “SurviveHF” to promote the key message of this paper within and beyond the heart failure community.

## Results

A total of 385 CPRD practices contributed data, linked to HES and ONS, between 1 January 2000 and 31 December 2017. Around 58% (n=411) of practices within CPRD are linked to HES data. We did not include practices that did not report data for the study period or had no eligible patients. There were 2 456 338 patients aged 45 and over who were registered for at least one year in the study period and were eligible for linkage. We identified 55 959 patients with incident heart failure and matched them to 278 679 controls (see appendix 2). In the heart failure group, 24 125 people (43.1%) were admitted to hospital around the time of diagnosis overall. The percentage of patients who required hospital admission around the time of diagnosis increased between 2000 and 2007, and then remained stable from 2008 onwards.

Table 1 shows the baseline characteristics of patients with heart failure and matched comparators (and by admission to hospital around the time of diagnosis and socioeconomic status in appendix 3). The average age at diagnosis was 77.1 (standard deviation 10.6) overall and did not change over the 18 year period. Women were on average almost five years older at diagnosis than men (79.6 *v* 74.8 years). The proportion of people with heart failure admitted to hospital around the time of diagnosis increased from 28.9% (n=1070) in 2000 to 51.8% (n=1613) in 2010,****and then remained stable. Women, older people, and patients who were most deprived group were admitted to hospital more often around the time of diagnosis. Cardiovascular comorbidity was common in the heart failure group overall, but did not vary by admission to hospital and index of multiple deprivation. Average blood pressure was lower in patients with heart failure than in the group without heart failure (systolic 137.5 *v* 139.6 mm Hg, diastolic 76.9 *v* 77.6 mm Hg). Hypertension is a major risk factor for heart failure and a history of hypertension was more common in patients with heart failure (57.7% *v* 46.9%). The lower blood pressure in the patients with heart failure could be due to antihypertensive treatment or a direct consequence of heart failure.

**Table 1:** Baseline characteristics of people with heart failure and matched comparators. Data are number (%) unless stated otherwise. SD=standard deviation

Characteristic	Heart failure (n=55 959)	No heart failure (n=278 679)
Sex:		
Male	29 234 (52.2)	145 552 (52.2)
Female	26 725 (47.8)	133 127 (47.8)
Age (years, mean (SD))	77.08 (10.6)	76.08 (10.4)
Age group (years):		
45-54	1938 (3.5)	11 056 (4.0)
55-64	5426 (9.7)	29 124 (10.5)
65-74	12 485 (22.3)	67 851 (24.3)
75-84	21 534 (38.5)	110 015 (39.5)
85-94	13 453 (24.0)	57 065 (20.5)
≥95	1123 (2.0)	3568 (1.3)
Ethnic group:		
White	44 143 (78.9)	204 936 (73.5)
Non-white	1497 (2.7)	6516 (2.3)
Mixed	6585 (11.8)	38 114 (13.7)
Missing	3734 (6.7)	29 113 (10.4)
Index of multiple deprivation (fifths):		
1 (least deprived)	10 854 (19.4)	60 020 (21.5)
2	12 954 (23.1)	68 200 (24.5)
3	11 947 (21.3)	58 956 (21.2)
4	11 707 (20.9)	53 778 (19.3)
5 (most deprived)	8447 (15.1)	37 450 (13.4)
Missing	50 (0.1)	75 (0.1)
Smoking status:		
Never	21 252 (38.0)	123 460 (44.3)
Former	7094 (12.7)	31 494 (11.3)
Current	24 507 (43.8)	100 719 (36.1)
Missing	3106 (5.6)	23 006 (8.3)
Systolic blood pressure:		
Mean (SD; mm Hg)	137.54 (21)	139.59 (18.0)
Missing	1272 (2.3)	14 367 (5.2)
Diastolic blood pressure:		
Mean (SD; mm Hg)	76.89 (11.6)	77.64 (10.0)
Missing	1272 (2.3)	14 367 (5.2)
Total cholesterol:		
Mean (SD; mmol/L)	4.69 (3.6)	5.03 (2.5)
Missing	15 478 (27.7)	104 041 (37.3)
Body mass index:		
Mean (SD)	27.93 (6.1)	26.53 (4.8)
Missing	8428 (15.1)	50 110 (18)
Medical history:		
Atrial fibrillation	14 629 (26.1)	20 910 (7.5)
Angina	11 965 (21.4)	29 508 (10.6)
Diabetes	13 104 (23.4)	38 824 (13.9)
Hypertension	32 316 (57.7)	130 669 (46.9)
Ischaemic heart disease	14 606 (26.1)	31 517 (11.3)
Myocardial infarction	11 296 (20.2)	17 537 (6.3)
Stroke	6271 (11.2)	19 409 (7.0)
Valve disease	4154 (7.4)	6165 (2.2)
Other cardiovascular disease	13 757 (24.6)	34 578 (12.4)

### Cause of death

Table 2 shows the cause of death for patients with heart failure and for comparators. There were 30 906 deaths in the heart failure group over the study period. The primary cause of death was heart failure in 2237 (7.2%) people, but listed as any cause in 13 093 (42.4%). Heart failure was also the primary cause of death in 960 (1.3%) of the no heart failure group. The second commonest cause of death for people with heart failure was respiratory disease (4925, 15.9%) followed by cancer (3854, 12.5%).

**Table 2:** Cause of death in people with heart failure and age, sex, and practice matched comparators without heart failure. Data are number (%)

Cause of death subgroup	Heart failure	No heart failure
Diseases of the circulatory system	17 207 (55.7)	24 965 (32.7)
Heart failure primary cause	2237 (7.2)	960 (1.3)
Heart failure any cause of death*	13 093 (42.4)	5528 (7.2)
Arrhythmias (ICD-10 codes: I47-I49)	513 (1.7)	684 (0.89)
Diseases of the respiratory system	4925 (15.9)	12 223 (16.0)
Neoplasms	3854 (12.5)	19 887 (26.0)
Diseases of the digestive system	1095 (3.5)	3526 (4.6)
Diseases of the genitourinary system	774 (2.5)	1877 (2.5)
Endocrine, nutritional, and metabolic diseases	611 (2.0)	876 (1.2)
Mental and behavioural disorders	484 (1.6)	4357 (5.7)
External causes of morbidity and mortality	438 (1.4)	1752 (2.3)
Symptoms, signs, and abnormal clinical and laboratory findings, not elsewhere classified	372 (1.2)	2263 (3.0)
Certain infectious and parasitic diseases	331 (1.1)	873 (1.1)
Diseases of the nervous system	318 (1.0)	2635 (3.5)
Diseases of the musculoskeletal system and connective tissue	219 (0.71)	689 (0.9)
Diseases of the skin and subcutaneous tissue	174 (0.6)	307 (0.4)
Diseases of the blood and blood-forming organs	63 (0.2)	144 (0.2)
Congenital malformations, deformations, and chromosomal abnormalities	36 (0.1)	44 (0.1)
Other	5 (0.0)	19 (0.0)

ICD-10=international classification of diseases, 10th revision.

* Includes patients for whom heart failure may have been the primary or a contributory cause of death.

### Survival in heart failure group

Survival rates in patients with heart failure were 75.9% (95% confidence interval 75.5% to 76.3%) at one year, 45.5% (45.1 to 46.0) at five years, 24.5% (23.9 to 25.0) at 10 years, and 12.7% (11.9 to 13.5) at 15 years. Table 3 shows survival rates by age and sex. Women had worse short term and long term outcomes than men (one year survival 74.5% *v* 77.2% (P<0.001) and 15 year survival 11.0% *v* 14.1% (P<0.001)). Age at diagnosis was a significant determinant of subsequent survival.

**Table 3:** Survival rates at one, five, 10, and 15 years after a diagnosis of heart failure overall and by sex and 10 year age group

Subgroup	Survival rate (% (95% CI))			
	At one year	At five years	At 10 years	At 15 years
Overall	75.9 (75.5 to 76.3)	45.5 (45.1 to 46.0)	24.5 (23.9 to 25.0)	12.7 (11.9 to 13.5)
Sex:				
Male	77.2 (76.7 to 77.7)	46.9 (46.3 to 47.6)	25.8 (25.0 to 26.5)	14.1 (13.1 to 15.2)
Female	74.5 (74.0 to 75.0)	44.0 (43.3 to 44.7)	23.0 (22.2 to 23.8)	11.0 (9.9 to 12.2)
Age group (years):				
45-54	90.3 (89.0 to 91.7)	78.5 (76.4 to 80.6)	64.7 (61.6 to 68.0)	54.4 (50.0 to 59.3)
55-64	87.9 (87.0 to 88.8)	70.6 (69.3 to 72.0)	52.8 (50.9 to 54.7)	38.4 (35.6 to 41.5)
65-74	83.5 (82.8 to 84.1)	59.1 (58.1 to 60.1)	35.4 (34.2 to 36.6)	17.2 (15.5 to 19.2)
75-84	76.5 (76.0 to 77.1)	43.2 (42.4 to 44.0)	18.4 (17.6 to 19.2)	5.8 (4.9 to 7.0)
85-94	63.2 (62.4 to 64.1)	22.3 (21.4 to 23.2)	4.4 (3.8 to 5.2)	0.2 (0.04 to 1.4)
≥95	43.9 (41.0 to 47.1)	6.0 (4.4 to 8.3)	—	—

### Trends in survival over time

**Fig 1:**
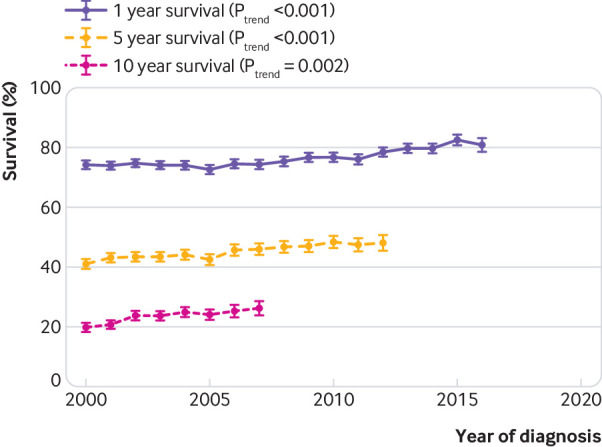
Survival rates at one, five, and 10 years for people with heart failure by year of diagnosis

Overall one year survival improved by 6.6% (95% confidence interval 4.0% to 9.2%) over time for people with a new diagnosis of heart failure from 74.2% (72.8% to 75.6%) in 2000 to 80.8% (78.6% to 83.1%) in 2016 (fig 1). Five year survival improved by 7.2% (4.2% to 10.2%) from 41.0% (39.4% to 42.7%) in 2000 to 48.2% (45.7% to 50.7%) in 2012. Ten year survival improved by 6.4% (3.6% to 9.1%) from 19.8% (18.4% to 21.3%) in 2000 to 26.2% (24.0% to 28.6%) in 2007. All trends remained when survival rates were standardised by age and sex, and across age groups.

### Survival in patients admitted to hospital at time of diagnosis

**Fig 2:**
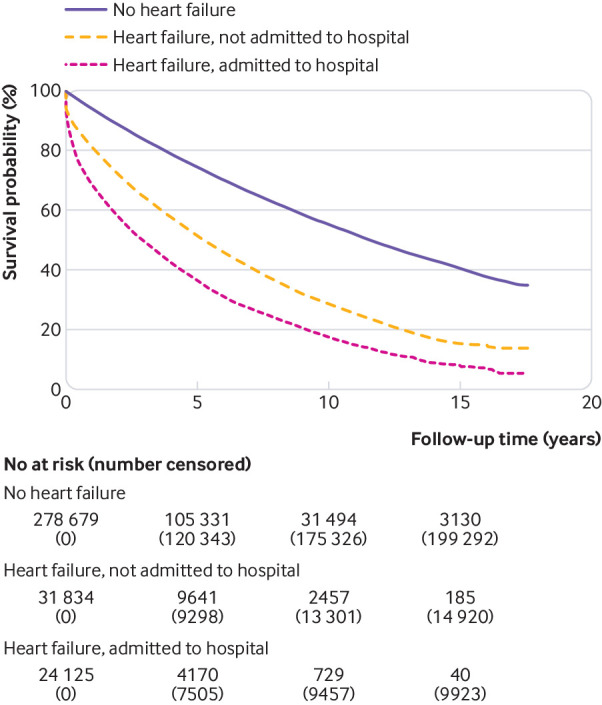
Kaplan-Meier curve of survival for people with a new diagnosis of heart failure who were admitted to hospital or not admitted to hospital at time of diagnosis and for comparators matched by age, sex, and practice

**Fig 3:**
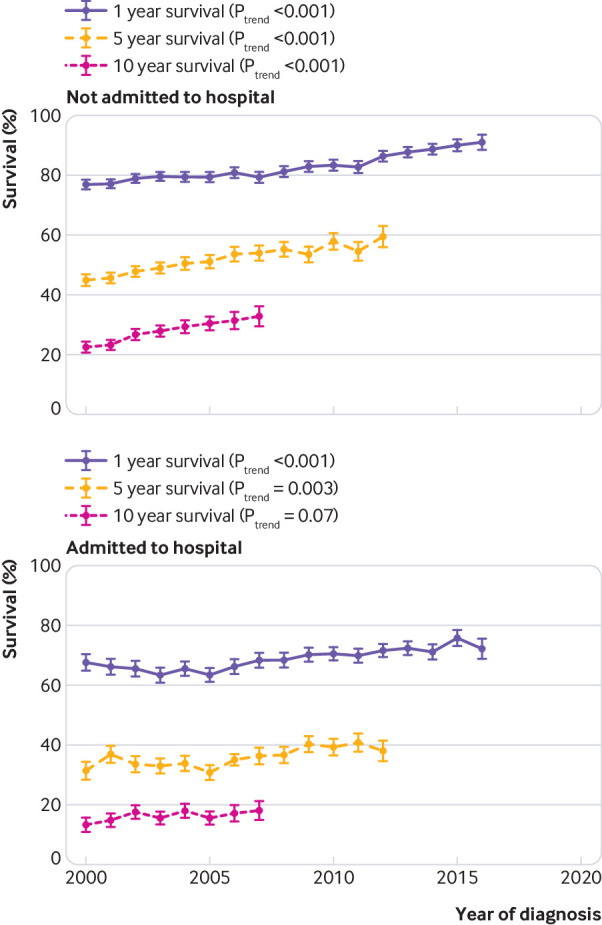
Survival at one, five, and 10 years in patients with heart failure not requiring hospital admission and requiring hospital admission around time of diagnosis by year of diagnosis

Survival of people with heart failure admitted to hospital around the time of diagnosis was significantly worse than in those not requiring hospital admission (fig 2), with a median difference of 2.4 years (5.3 *v* 2.9 years, log rank test, P<0.001). One year survival was 81.2% versus 68.8%, five year survival was 51.8% versus 36.7%, 10 year survival was 28.8% versus 17.8%, and 15 year survival was 15.5% versus 8.1% for patients not admitted to hospital and admitted to hospital, respectively. Survival rates by year of diagnosis improved more rapidly for people whose condition was diagnosed and managed in the community than for people requiring hospital admission at the time of diagnosis (fig 3).

### Socioeconomic inequalities

**Fig 4:**
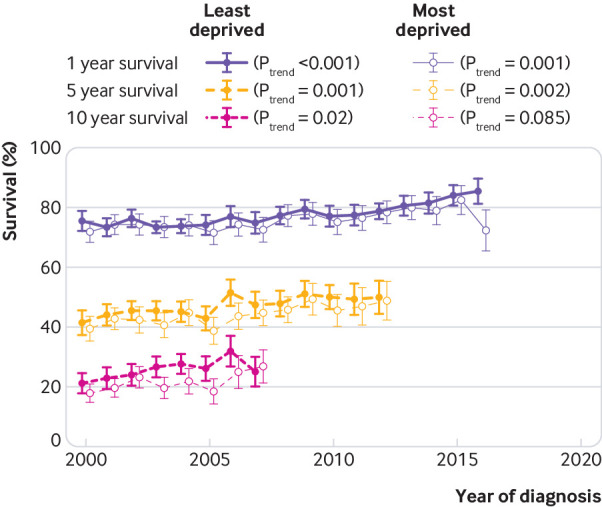
Trends in one, five, and 10 year survival by the least deprived and most deprived group****

In patients with heart failure, there were 10 854 in the least deprived group and 8447 in the most deprived group. Overall, there was a deprivation gap of 0.5 years in median survival between the least deprived and most deprived groups (4.6 *v* 4.1 years, P<0.001). Figure 4 shows the trends in survival over time by deprivation group. There was little difference between one and five year survival in the most deprived and least deprived groups over the study period, but 10 year survival was lower in the most deprived group from 2000 to 2006, although this gap improved in 2007. A mixed effects Cox model, adjusting for age, sex, year of diagnosis, and practice (cluster effect), indicated that the risk of death increased by 6% with level of deprivation (hazard ratio 1.06, 95% confidence interval 1.05 to 1.07).

### Overall survival

**Fig 5:**
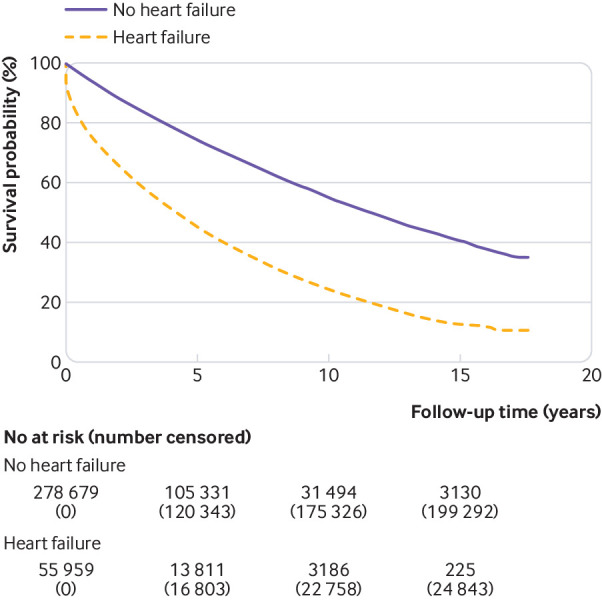
Kaplan-Meier curve of overall survival for people with a new diagnosis of heart failure and comparators matched by age, sex, and practice

The median time to death in those patients with heart failure who died during the study period was 18 months compared with 36 months in those without heart failure who died during the study period. Cox regression analysis suggested that overall patients with heart failure had a significantly worse prognosis than their age, sex, and practice matched comparators, as shown in figure 5 (hazard ratio 3.36, 95% confidence interval 3.31 to 3.42). An increased risk of death remained for patients with heart failure after adjustment for deprivation, ethnicity, and cardiovascular risk factors (3.05, 3.00 to 3.11). We observed similar results in the sensitivity analysis of the fully adjusted model (3.17, 3.09 to 3.26) from a complete case analysis of 186 285 patients; and in the adjusted model in which cholesterol and body mass index were excluded (3.02, 2.96 to 3.08, with 280 211 patients).

## Discussion

### Principal findings

This large community based study provides contemporary survival estimates for people with a new diagnosis of heart failure across an 18 year period. People admitted to hospital around the time of diagnosis had worse survival rates than those whose condition was diagnosed and managed in the community. Survival after heart failure diagnosis gradually improved over time, but more rapidly in the group not admitted to hospital. The deprivation gap in survival of 0.5 years between the least deprived and most deprived groups suggests socioeconomic inequalities in heart failure care.

### Strengths and limitations of study

We identified a total of 55 959 people with a new diagnosis of heart failure from 2000 to the end of 2017, which allowed reliable analyses of subgroups (year of diagnosis, hospital admission, and socioeconomic status) and recent survival trends. The data were taken directly from primary and secondary care sources with linkage between databases. ONS mortality data are determined by the information provided on the death certificate by the doctor caring for the patient around the time of death. Although we are aware that death recording in general practice can vary and death certification may be inaccurate in some cases, ONS data are the most reliable source available.^23^ ^27^

In the UK, the entire population receives healthcare through registration with a primary care provider, and routinely collected data are being increasingly used to explore epidemiological trends.^28^ ^29^ The main reason for primary care physicians to code medical information is to provide clinical care rather than for research purposes, and this can potentially lead to incomplete data. However, previous studies have shown that recording of diagnoses in CPRD is good,^30^ as is recording of heart failure in healthcare databases more generally.^31^ We also tried to confirm primary care diagnoses using hospital data. Any under-reporting of heart failure would have diluted the observed association between heart failure and mortality, so we are confident that our overall findings are robust.

Practices using the Vision clinical system provide data directly to CPRD. In the past few years, CPRD has seen a reduction in the number of practices contributing data because of a decline in the popularity of Vision software. There is a move for CPRD to link with alternative clinical system providers to maintain the large dataset. Despite the lower number of practices providing data towards the end of the study period and a moderate level of unrecorded data being observed, our sensitivity analysis suggests our conclusions are robust.

We realise that people with pre-existing heart failure might be misclassified as having a new diagnosis of heart failure or not having the condition; however this misclassification is likely to be minimised by linking electronic data from CPRD with HES and ONS mortality data.^32^ Evidence of close agreement between heart failure incidence in CPRD with population surveillance studies has also been found.^33^ We were unable to identify the type of heart failure (reduced or preserved ejection fraction) in this study, which would have been desirable because of the differences in management depending on ejection fraction. We hope this will be possible in the future when echocardiography coding becomes more sophisticated and codes for heart failure with preserved ejection are increasingly used.

Heart failure was listed as a cause of death in less than half of the people with the condition. Other cardiovascular diseases, respiratory disorders, and cancer were also common causes of death in people diagnosed as having heart failure. People with heart failure are often living with several long term conditions; a large study using UK data found that 79% of people with heart failure had three or more comorbidities.^4^ Cardiovascular diseases, particularly atrial fibrillation, diabetes, ischaemic heart disease, and stroke, were the most common comorbidities. Other diseases including cancer (25%), chronic kidney disease (24%), and depression (22%) were prevalent and comorbidities occurred at a younger age in deprived populations with heart failure. In the no heart failure group, heart failure was listed as the primary cause of death in 1.3%. This finding probably relates to people who developed acute heart failure and died soon afterwards; therefore, they did not have a diagnosis in their primary care record. In the most recent National Heart Failure audit, inhospital mortality was 9.4%.^10^

Multimorbidity (two or more long term conditions) can make the diagnosis of heart failure difficult because symptoms overlap and management is more challenging. Two thirds of people with heart failure have three or more other long term conditions.^4^ In our study, the average age of diagnosis in primary care was 77, therefore survival after diagnosis is unlikely to depend on heart failure care alone. Holistic, person centred care is required to optimally manage people with heart failure in the context of multimorbidity, with a focus on quality of life as well as length of survival.

Our study did not explore the effect of drugs, devices, or transplantation on the survival of people with heart failure. The aim was to present contemporary short term, mid-term, and long term survival rates at a population level, and to explore trends over time. Further research exploring new treatments, technologies, guidelines, and health policies is also needed to understand their impact at the patient level.

### Comparison with other studies

There is considerable variation in previous heart failure survival estimates depending on the study setting. People with acute heart failure recruited from hospital inpatient populations have comparatively poor outcomes. Among 12 440 people with heart failure in the European Society of Cardiology Heart Failure Long-Term registry, one year mortality was 23.6% for people with acute heart failure and 6.4% for those with chronic heart failure across Europe.^34^ Despite initiatives in many countries to improve care for people with heart failure, success has been limited. The most recent annual national heart failure audit in England reported one year mortality rates of 29.6% among people admitted to hospital with heart failure. This rate had not improved for the previous six years despite service restructuring to provide patients with care from specialist heart failure teams during admission and immediately after discharge.^10^

Population based studies, such as the Framingham or the Echocardiographic Heart of England Screening study, have reported long term survival for people with heart failure, but participants were invited for screening rather than presenting to primary care with symptoms.^11^ ^35^ In the Echocardiographic Heart of England Screening study cohort, five and 10 year survival rates of people with heart failure were 53% and 27%, but diagnosis at screening could represent an earlier disease stage.^35^ Several European countries have established registries using routinely collected healthcare data to monitor trends in long term conditions. In Sweden, among 88 038 people with heart failure, overall five year survival was 48% and survival rates improved by 19% between 2006 and 2010.^14^

Our previous study explored survival rates in The Health Improvement Network database between 1998 and 2012, without HES or index of multiple deprivation linkage, and found no improvement in survival over that period.^13^ This conclusion was of concern to the heart failure community and we wanted to conduct a further analysis using a different dataset linked to hospital and deprivation data to verify and explore the findings. In this CPRD study, we have provided contemporary survival rates up to 2017, which have shown a modest improvement since 2000. We have also been able to analyse survival related to hospital admission around the time of diagnosis and among socioeconomic groups. This analysis has highlighted the importance of timely diagnosis in primary care and the gap in survival between the most deprived and least deprived groups.

### Conclusions and policy implications

In this study we found gradual improvements in survival rates over time, which is encouraging. However, the outlook after a new diagnosis of heart failure, particularly for those requiring admission to hospital, remains poor. Hospital admission at the time of diagnosis probably relates to a more advanced stage of disease. Lead time bias might also be a contributory factor; patients with an earlier diagnosis appear to live longer. Earlier diagnosis in primary care, however, does allow treatment initiation, potentially avoiding emergency admission to hospital and improving patient outcomes.

Heart failure has not been a priority area in government policy or funding, and other serious conditions, such as cancer, have seen a much greater improvement in survival over time.^36^ However, in 2006 a heart failure indicator was introduced to the Quality and Outcomes Framework for general practitioners in England. The indicator aimed to incentivise general practitioners to use echocardiography to support diagnosis and initiation of angiotensin converting enzyme inhibitors and β blockers for people with an ejection fraction below 40%. We could not establish the direct effect of this indicator on our data, but the 10 year survival difference between the least deprived and most deprived groups, observed in previous years, became non-significant in 2007. Future work is needed to monitor the trends in long term survival since the new indicator was introduced.

There has been a sustained improvement in cancer survival rates following the introduction of the Cancer Plan in 2000, which included investment and infrastructure changes to improve diagnosis and treatment.^37^ The lack of substantial progress in improving heart failure survival rates should alert policy makers to the need for further investment in heart failure services. Improved general practitioners access to diagnostics such as natriuretic peptide testing, rapid referral pathways (such as the “two week wait cancer” pathways) for echocardiography, and specialist assessment and early treatment initiation might be areas for improvement. Primary care led research is also needed to understand the complexity of heart failure diagnosis and management in the community, and to develop and test new strategies to achieve better outcomes for patients.

**What is already known on this topic**

Heart failure is an increasingly prevalent condition that affects over 920 000 people in the UK
Survival for people with established heart failure is poor, and studies exploring survival trends over time are inconsistent

**What this study adds**

This study provides estimates of short term and long term survival rates for people with a new diagnosis of heart failure in primary care
Improvement in survival since 2000 has been modest and socioeconomic inequalities persist
People who did not require hospital admission around the time of diagnosis lived longer, which could reflect detection at an earlier stage of disease

## Web extra

Extra material supplied by authors

**Web appendix 1:** Codes to identify heart failure in CPRD

**Web appendix 2:** Study flowchart

**Web appendix 3:** Supplementary table

## Notes

Contributors: CJT, FDRH, and TM conceived the study. CJT, AKR, and SL-F wrote the protocol. SL-F extracted the data from the Clinical Practice Research Datalink (CPRD). JMOM carried out the statistical analysis. AKR provided guidance on statistical analysis. NRJ conducted the literature search. TM provided critical input to the analysis plan. All authors contributed to the interpretation of results and revisions of the manuscript. CJT wrote the manuscript and is the guarantor. CJT and FDRH are joint corresponding authors. The corresponding authors attest that all listed authors meet authorship criteria and that no others meeting the criteria have been omitted.

Ethical approval: The full study protocol was approved by the independent scientific advisory committee (ISAC) to the Medicine and Healthcare products Regulatory Authority (protocol number 18_061R). Ethical approval for observational research using CPRD with approval from ISAC has been granted by a national research ethics service committee (05/MRE04/87).

Data sharing: This study is based in part on data from CPRD obtained under licence from the UK Medicines and Healthcare products Regulatory Agency. The data are provided by patients and collected by the NHS as part of their care and support. The Office for National Statistics provided the mortality data. The interpretation and conclusions contained in this study are those of the authors alone. The authors do not own these data and hence are not permitted to share the data in the original form.

The lead author (CJT) affirms that the manuscript is an honest, accurate, and transparent account of the study being reported; that no important aspect of the study has been omitted; and that any discrepancies from the study as planned have been explained.
