# Trends in survival after a diagnosis of heart failure in the United Kingdom 2000-2017: population based cohort study

**DOI:** 10.1136/bmj.l5840

**Published:** 2019-10-08

In this paper by Taylor and colleagues (*BMJ* 2019;364:l223, doi:10.1136/bmj.l223, published 13 February 2019), the authors identified the following errors after reanalysis of the dataset:

- In the overall survival subsection of the results, the first sentence incorrectly mentioned median survival time instead of median time to death in patients who died during the study period. The sentence should have stated: “The median time to death in those patients with heart failure who died during the study period [not “The median survival for patients with heart failure”] was 18 months compared with 36 months in those without heart failure who died during the study period [not “was 18 months compared with 36 months for those without heart failure”].”
- In the results paragraph of the abstract, the last sentence should read: “There was a deprivation gap in median survival of 0.5 years between people who were least deprived and those who were most deprived (4.6 *v* 4.1 years, P<0.001).”
- In the results section, the second sentence of the socioeconomic inequalities paragraph should read: “Overall, there was a deprivation gap of 0.5 years in median survival between the least deprived and most deprived groups (4.6 *v* 4.1 years, P<0.001).”
- In the results section, the last sentence of the socioeconomic inequalities paragraph should read: “A mixed effects Cox model, adjusting for age, sex, year of diagnosis, and practice (cluster effect), indicated that the risk of death increased by 6% with level of deprivation (hazard ratio 1.06, 95% confidence interval 1.05 to 1.07).”
- In the discussion section, the last sentence of the principal findings paragraph should read: “The deprivation gap in survival of 0.5 years between the least deprived and most deprived groups suggests socioeconomic inequalities in heart failure care.”

The article will be updated in due course.

