# Supplementary material for: Trends in survival after a diagnosis of heart failure in the United Kingdom 2000-2017: population based cohort study
Source: BMJ. 2019 Feb 13;364:l223. doi: 10.1136/bmj.l223 (PMC6372921; doi:10.1136/bmj.l223)
Supplement: Supplementary file 1 — Web appendix 1: Codes to identify heart failure in CPRD [file tayc047415.ww1.pdf]

Codes to identify heart failure in CPRD

Medical code	Read code	Read term
9913	1O1..00	Heart failure confirmed
21837	G232.00	Hypertensive heart&renal dis wth (congestive) heart failure
2062	G58..00	Heart failure
1223	G58..11	Cardiac failure
398	G580.00	Congestive heart failure
2906	G580.11	Congestive cardiac failure
10079	G580.12	Right heart failure
10154	G580.13	Right ventricular failure
9524	G580.14	Biventricular failure
23707	G580000	Acute congestive heart failure
32671	G580100	Chronic congestive heart failure
27884	G580200	Decompensated cardiac failure
11424	G580300	Compensated cardiac failure
94870	G580400	Congestive heart failure due to valvular disease
884	G581.00	Left ventricular failure
43618	G581.12	Pulmonary oedema - acute
5255	G581000	Acute left ventricular failure
27964	G582.00	Acute heart failure
101138	G583.00	Heart failure with normal ejection fraction
101137	G583.11	HFNEF - heart failure with normal ejection fraction
106897	G583.12	Heart failure with preserved ejection fraction
104275	G584.00	Right ventricular failure
4024	G58z.00	Heart failure NOS
17278	G58z.12	Cardiac failure NOS
96799	G5y4z00	Post cardiac operation heart failure NOS
66306	SP11111	Heart failure as a complication of care

Codes to identify heart failure in hospital records

ICD code	Term
I50	Heart failure
I50.0	Congestive heart failure
I50.1	Left ventricular failure
I50.9	Heart failure, unspecified
I11.0	Hypertensive heart disease with (congestive) heart failure
I13.0	Hypertensive heart and renal disease with (congestive) heart failure
I13.2	Hypertensive heart and renal disease with both (congestive) heart failure and renal failure

Heart failure codes indicating preserved ejection fraction

Medical code	Read code	Read term
12314	585k.00	Echocardiogram shows normal left ventricular function
101138	G583.00	Heart failure with normal ejection fraction
101137	G583.11	HFNEF - heart failure with normal ejection fraction
106897	G583.12	Heart failure with preserved ejection fraction

Codes to identify echocardiography in CPRD

Medical code	Read code	Read term
1271	5853.11	Echocardiogram
23268	5853	U-S heart scan
1432	5853000	Echocardiogram normal
5245	5853100	Echocardiogram abnormal
11284	585f.00	Echocardiogram shows left ventricular systolic dysfunction
18508	585R.00	Echocardiogram normal
26886	33BD.00	Echocardiogram requested
11351	585g.00	Echocardiogram shows left ventricular diastolic dysfunction
12314	585k.00	Echocardiogram shows normal left ventricular function
27851	5853z00	U-S heart scan NOS
30917	5C20.00	Echocardiogram equivocal
10317	R132000	[D]Echocardiogram abnormal
11683	8HQ7.00	Referral for echocardiography
3919	7935200	Transoesophageal echocardiography
39134	7P0H400	Stress echocardiography
89353	7935500	Transluminal intracardiac echocardiography
26449	7P0H.00	Diagnostic echocardiography
26445	7P0H000	Transthoracic echocardiography
94626	7P0H300	Epicardial echocardiography
90493	7P0Hz00	Diagnostic echocardiography NOS
105649	8A54400	Monitoring of cardiac output using echocardiography
105899	8A58.00	Transoesophageal echocardiographic monitoring
102428	8H7o000	Fast track HF referral for transthoracic 2D echocardiogram
101184	9Ee0800	Adult echocardiography procedure report
106377	9EV7.00	Echocardiography report received
99841	7P0Hy00	Other specified diagnostic echocardiography
105341	7P0H600	Contrast echocardiography
85952	7P0H100	Transoesophageal echocardiography

Codes to identify echocardiography in hospital records

OPCS code	Term
U200	Diagnostic echocardiography
U201	Transthoracic echocardiography
U202	Transoesophageal echocardiography
U203	Intravascular echocardiography
U204	Epicardial echocardiography
U205	Stress echocardiography
U208	Other specified diagnostic Echocardiography
U209	Unspecified diagnostic Echocardiography
