# Supplementary figures and images for: Trends in survival after a diagnosis of heart failure in the United Kingdom 2000-2017: population based cohort study
Source: BMJ. 2019 Feb 13;364:l223. doi: 10.1136/bmj.l223 (PMC6372921; doi:10.1136/bmj.l223)

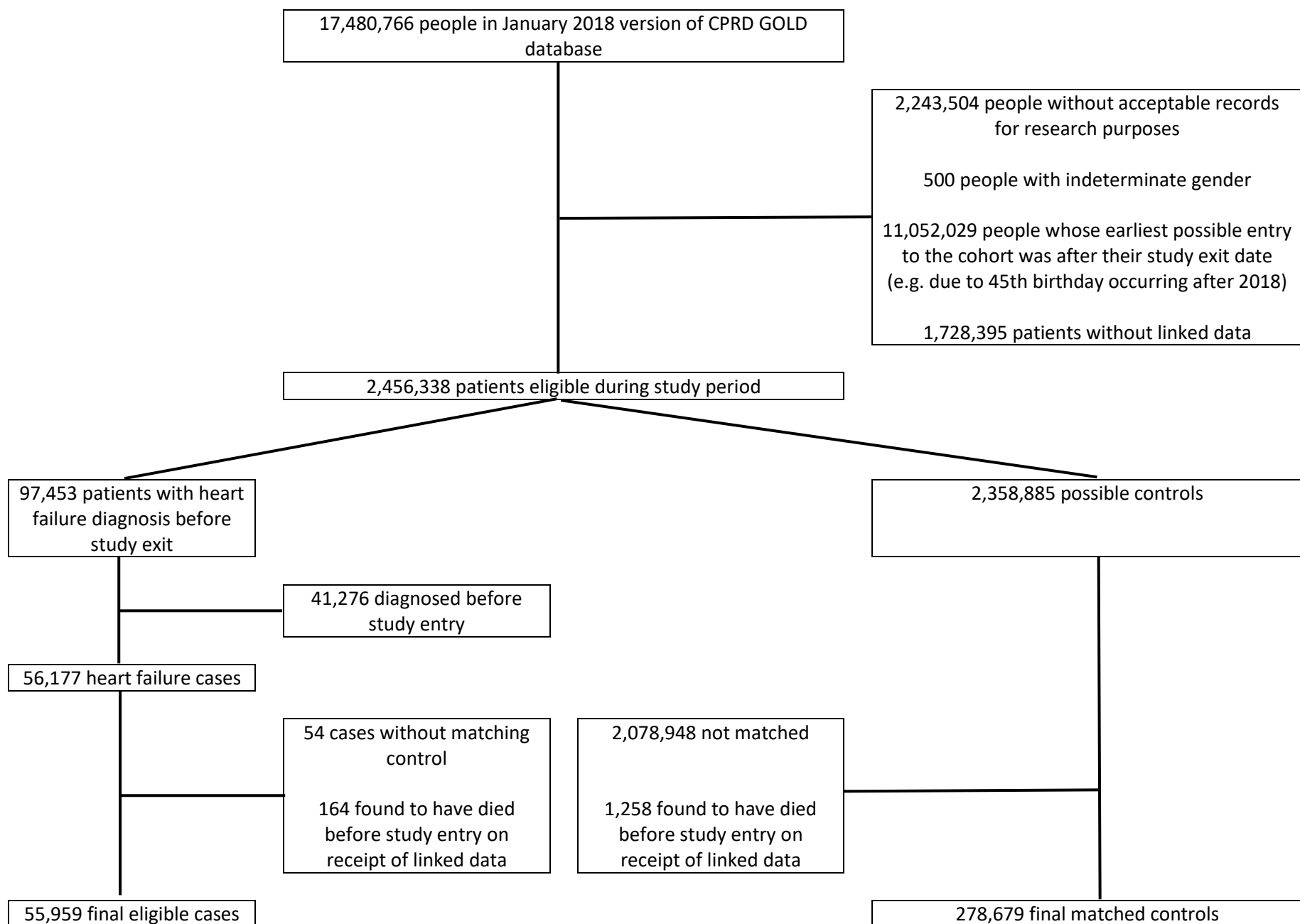

Supplement: Supplementary file 2 — Web appendix 2: Study flowchart [file tayc047415.ww2.pdf]
