# Supplementary material for: Trends in survival after a diagnosis of heart failure in the United Kingdom 2000-2017: population based cohort study
Source: BMJ. 2019 Feb 13;364:l223. doi: 10.1136/bmj.l223 (PMC6372921; doi:10.1136/bmj.l223)
Supplement: Supplementary file 3 — Web appendix 3: Supplementary table [file tayc047415.ww3.pdf]

Table 1: Baseline characteristics of people with heart failure and matched comparators and, in the heart failure group, by hospitalisation and socioeconomic status

	Cohort				Heart Failure				Heart Failure			
	Heart Failure		No Heart Failure		Hospitalisation around time of diagnosis				Index of Multiple Deprivation quintile			
**Characteristic**					Yes		No		Least deprived		Most deprived	
	**n**	**%**	**n**	**%**	**n**	**%**	**n**	**%**	**n**	**%**	**n**	**%**
**Overall, n (%)**	55,959	(100)	278,679	(100)	24,125	(43.1)	31,834	(56.9)	10,854	(19.4)	8,447	(15.1)
**Sex, n (%)**												
Male	29,234	(52.2)	145,552	(52.2)	12,438	(42.5)	16,796	(57.5)	5,885	(20.1)	4,282	(14.6)
Female	26,725	(47.8)	133,127	(47.8)	11,687	(43.7)	15,038	(56.3)	4,969	(18.6)	4,165	(15.6)
**Age, mean (SD)**	77.08	(10.6)	76.08	(10.4)	77.62	(10.7)	76.67	(10.4)	78.24	(10.2)	74.86	(11)
**Age category, n (%)**												
45-54	1,938	(3.5)	11,056	(4.0)	855	(44.1)	1,083	(55.9)	268	(13.8)	444	(22.9)
55-64	5,426	(9.7)	29,124	(10.5)	2,231	(41.1)	3,195	(58.9)	895	(16.5)	1,121	(20.7)
65-74	12,485	(22.3)	67,851	(24.3)	5,018	(40.2)	7,467	(59.8)	2,182	(17.5)	2,141	(17.1)
75-84	21,534	(38.5)	110,015	(39.5)	9,057	(42.1)	12,477	(57.9)	4,317	(20.0)	3,082	(14.3)
85-94	13,453	(24.0)	57065	(20.5)	6,405	(47.6)	7,048	(52.4)	2,931	(21.8)	1,549	(11.5)
95+	1,123	(2.0)	3,568	(1.3)	559	(49.8)	564	(50.2)	261	(23.2)	110	(9.8)
**Ethnic group, n (%)**												
White	44,143	(78.9)	204,936	(73.5)	19,547	(44.3)	24,596	(55.7)	8,643	(19.6)	6,574	(14.9)
Non-white	1,497	(2.7)	6,516	(2.3)	694	(46.4)	803	(53.6)	194	(13.0)	359	(24.0)
Mixed	6,585	(11.8)	38,114	(13.7)	2,970	(45.1)	3,615	(54.9)	1,194	(18.1)	1,026	(15.6)
Missing	3,734	(6.7)	29,113	(10.4)	914	(24.5)	2,820	(75.5)	823	(22.0)	488	(13.1)
**Index of multiple deprivation (quintile)**												
1 (least deprived)	10,854	(19.4)	60,020	(21.5)	4,360	(40.2)	6,494	(59.8)	10,854	(100)		
2	12,954	(23.1)	68,200	(24.5)	5,526	(42.7)	7,428	(57.3)				
3	11,947	(21.3)	58,956	(21.2)	5,099	(42.7)	6,848	(57.3)				
4	11,707	(20.9)	53,778	(19.3)	5,250	(44.8)	6,457	(55.2)				
5 (most deprived)	8,447	(15.1)	37,450	(13.4)	3,866	(45.8)	4,581	(54.2)			8,447	(100)
Missing	50	(0.1)	75	(0.1)	24	(0.1)	52	(0.1)				
**Smoking status, n (%)**												
Never	21,252	(38.0)	123,460	(44.3)	8,879	(41.8)	12,373	(58.2)	4,711	(22.2)	2,589	(12.2)
Former	7,094	(12.7)	31,494	(11.3)	3,255	(45.9)	3,839	(54.1)	930	(13.1)	1,756	(24.8)
Current	24507	(43.8)	100,719	(36.1)	10917	(44.5)	13,590	(55.5)	4,661	(19.0)	3,639	(14.8)

Missing	3,106	(5.6)	23,006	(8.3)	1,074	(34.6)	2,032	(65.4)	552	(17.8)	463	(14.9)
**SBP, n (%)**												
Mean (SD) (mmHg)	137.54	(21)	139.59	(18.0)	136.18	(21.2)	138.58	(20.7)	137.2	(20.7)	137.92	(21.1)
Missing	1,272	(2.3)	14,367	(5.2)	426	(33.5)	846	(66.5)	287	(22.6)	201	(15.8)
**DBP, n (%)**												
Mean(SD) (mmHg)	76.89	(11.6)	77.64	(10.0)	76.24	(11.9)	77.39	(11.3)	76.96	(11.5)	77.23	(11.8)
Missing	1,272	(2.3)	14,367	(5.2)	426	(33.5)	846	(66.5)	287	(22.6)	201	(15.8)
**Total cholesterol, n (%)**												
Mean(SD)(mmol/L)	4.69	(3.6)	5.03	(2.5)	4.66	(5.2)	4.72	(1.2)	4.66	(1.2)	4.64	(1.2)
Missing	15,478	(27.7)	104,041	(37.3)	5,801	(37.5)	9,677	(62.5)	2,939	(19.0)	2,320	(15.0)
**BMI, n (%)**												
Mean(SD)(kg/m2)	27.93	(6.1)	26.53	(4.8)	27.98	(6.3)	27.88	(5.9)	27.31	(5.4)	28.6	(6.6)
Missing	8,428	(15.1)	50,110	(18)	3,356	(39.8)	5,072	(60.2)	1,610	(19.1)	1,272	(15.1)
**Medical History**												
**AF, n (%)**	14,629	(26.1)	20,910	(7.5)	6,554	(44.8)	8,075	(55.2)	3,094	(21.1)	1,930	(13.2)
**Angina, n (%)**	11,965	(21.4)	29,508	(10.6)	5,116	(42.8)	6,849	(57.2)	2,091	(17.5)	1,993	(16.7)
**Diabetes, n (%)**	13,104	(23.4)	38,824	(13.9)	6,589	(50.3)	6,515	(49.7)	2,198	(16.8)	2,315	(17.7)
**Hypertension n (%)**	32,316	(57.7)	130,669	(46.9)	14,623	(45.3)	17,693	(54.7)	6,260	(19.4)	4,873	(15.1)
**IHD, n (%)**	14,606	(26.1)	31,517	(11.3)	6,280	(43.0)	8,326	(57.0)	2,614	(17.9)	2,500	(17.1)
**MI, n (%)**	11,296	(20.2)	17,537	(6.3)	5,223	(46.2)	6,073	(53.8)	2,111	(18.7)	1,825	(16.2)
**Stroke, n (%)**	6,271	(11.2)	19,409	(7.0)	2,964	(47.3)	3,307	(52.7)	1,175	(18.7)	990	(15.8)
**VHD, n (%)**	4,154	(7.4)	6,165	(2.2)	2,097	(50.5)	2,057	(49.5)	937	(22.6)	466	(11.2)
**Other CVD, n (%)**	13,757	(24.6)	34,578	(12.4)	6,051	(44.0)	7,706	(56.0)	2,750	(20.0)	2,008	(14.6)
**Year, n (%)**												
2000	3,698	(6.6)	18,437	(6.6)	1,070	(28.9)	2,628	(71.1)	660	(17.8)	665	(18.0)
2001	4,368	(7.8)	21,771	(7.8)	1,257	(28.8)	3,111	(71.2)	783	(17.9)	741	(17.0)
2002	4,312	(7.7)	21,465	(7.7)	1,324	(30.7)	2,988	(69.3)	836	(19.4)	631	(14.6)
2003	4,337	(7.8)	21,599	(7.8)	1,478	(34.1)	2,859	(65.9)	857	(19.8)	628	(14.5)
2004	3,830	(6.8)	19,061	(6.8)	1,474	(38.5)	2,356	(61.5)	730	(19.1)	608	(15.9)
2005	3,550	(6.3)	17,675	(6.3)	1,502	(42.3)	2,048	(57.7)	683	(19.2)	528	(14.9)
2006	3,128	(5.6)	15,583	(5.6)	1358	(43.4)	1,770	(56.6)	579	(18.5)	503	(16.1)
2007	3,090	(5.5)	15,365	(5.5)	1,418	(45.9)	1,672	(54.1)	558	(18.1)	483	(15.6)
2008	3,121	(5.6)	15,516	(5.6)	1,451	(46.5)	1670	(53.5)	564	(18.1)	514	(16.5)
2009	3,265	(5.8)	16,261	(5.8)	1,621	(49.6)	1,644	(50.4)	633	(19.4)	468	(14.3)
2010	3,114	(5.6)	15,510	(5.6)	1,613	(51.8)	1,501	(48.2)	574	(18.4)	428	(13.7)
2011	2994	(5.4)	14,933	(5.4)	1,576	(52.6)	1,418	(47.4)	562	(18.8)	440	(14.7)

2012	3,189	(5.7)	15,867	(5.7)	1,730	(54.2)	1,459	(45.8)	638	(20.0)	492	(15.4)
2013	2,963	(5.3)	14,753	(5.3)	1,557	(52.5)	1,406	(47.5)	586	(19.8)	420	(14.2)
2014	2,648	(4.7)	13,191	(4.7)	1,371	(51.8)	1,277	(48.2)	565	(21.3)	323	(12.2)
2015	2,164	(3.9)	10,781	(3.9)	1,147	(53.0)	1,017	(47.0)	476	(22.0)	287	(13.3)
2016	1,503	(2.7)	7,493	(2.7)	815	(54.2)	688	(45.8)	363	(24.2)	190	(12.6)
2017	685	(1.2)	3,418	(1.2)	363	(53.0)	322	(47.0)	207	(30.2)	98	(14.3)

Abbreviations: AF: Atrial Fibrillation; BMI: Body Mass Index; CVD: Cardiovascular disease; DBP: Diastolic Blood Pressure; IHD: Ischaemic Heart Disease; MI: Myocardial Infarction; SBP: Systolic Blood Pressure; VHD: Valvular Heart Disease.
